# Preclinical testing of an Atr inhibitor demonstrates improved response to standard therapies for esophageal cancer

**DOI:** 10.1016/j.radonc.2016.10.023

**Published:** 2016-11

**Authors:** Katarzyna B. Leszczynska, Greg Dobrynin, Rhea E. Leslie, Jonathan Ient, Adam J. Boumelha, Joana M. Senra, Maria A. Hawkins, Tim Maughan, Somnath Mukherjee, Ester M. Hammond

**Affiliations:** Cancer Research UK and Medical Research Council Oxford Institute for Radiation Oncology, Department of Oncology, The University of Oxford, UK

**Keywords:** ATR, Esophageal cancer, Radiation, Hypoxia

## Abstract

**Background and purpose:**

Esophageal cancer has a persistently low 5-year survival rate and has recently been classified as a cancer of unmet need by Cancer Research UK. Consequently, new approaches to therapy are urgently required. Here, we tested the hypothesis that an ATR inhibitor, VX-970, used in combination with standard therapies for esophageal cancer could improve treatment outcome.

**Material and methods:**

Using esophageal cancer cell lines we evaluated the efficacy of combining VX-970 with cisplatin and carboplatin *in vitro* and with radiation *in vitro* and *in vivo*. Radiation experiments were also carried out in hypoxic conditions to mimic the tumor microenvironment.

**Results:**

Combining VX-970 with cisplatin, carboplatin and radiation increased tumor cell kill *in vitro*. A significant tumor growth delay was observed when VX-970 was combined with radiotherapy *in vivo*.

**Conclusions:**

VX-970 is an effective chemo/radiosensitizer which could be readily integrated in the current treatment paradigm to improve the treatment response in esophageal cancer and we plan to test it prospectively in the forthcoming phase I dose escalation safety study combining the ATR inhibitor VX-970 with chemoradiotherapy in esophageal cancer (EudraCT number: 2015-003965-27).

Esophageal cancer has been identified by CRUK as a cancer of unmet need, due to a persistent low 5-year survival rate (13%). In 2010, 8500 people were diagnosed with esophageal cancer in the UK and in 2011 there were 7600 deaths, making it the 4th most common cause of death in males. In recent decades the incidence of esophageal cancer has risen in western populations and there has been a shift in histology from squamous cell carcinoma (SCC) to adenocarcinoma (ACA). Tumors are commonly localized to the lower esophagus and gastro-esophageal junction. At presentation the majority of patients are unsuitable for surgery and have locally advanced or metastatic disease irrespective of histologic type. Platinum-based chemotherapy regimens (cisplatin, carboplatin, oxaliplatin) are the mainstay of treatment for esophageal cancer. Definitive chemo-radiotherapy (dCRT) is a treatment option in patients with localized inoperable disease and pre-operative (neo-adjuvant) chemoradiotherapy (nCRT) is increasingly becoming an established standard worldwide for operable patients. However, cures remain elusive and there is an urgent need to develop agents that can enhance the outcomes from the currently available treatment options [1].

ATR (ATM and rad 3-related), is an essential phosphatidylinositol 3-kinase (PI3K)-like kinase which responds to replication stress and DNA damage by initiating extensive downstream signaling to protect the cell from genome instability [2]. Once activated ATR can induce cell cycle arrest, inhibit replication origin firing, protect stalled replication forks and initiate DNA repair. An ATR inhibitor can potentiate the anti-cancer activity of DNA damaging agents (which include cisplatin, carboplatin and radiation). Recently, potent selective inhibitors of ATR (as opposed to the broad-spectrum inhibitors of other PI3K-related kinases) have been developed [3], [4]. There is a strong scientific rationale for combining ATR inhibitors with platinum based chemotherapy and radiation, the standard therapies for esophageal cancer, although *in vitro* and *in vivo* experiments using esophageal cell lines have not been previously reported. In other tumor sites, concurrent ATR inhibition has been shown *in vitro* to improve the response to cisplatin and radiation, both of which are key therapeutics in the treatment of esophageal cancer [5], [6]. In addition, ATR inhibition has been shown to be particularly cytotoxic to tumor cells with a deficiency in the ATM or p53 pathway [3], [7]. Esophageal cancers have a high incidence of p53 mutations (∼89.9% in SCC of the esophagus and ∼72% in ACA) [8]. Finally, regions of tumor hypoxia occur within esophageal cancers and HIF-1α overexpression has been shown to correlate with chemoresistance and poor patient prognosis [9], [10], [11], [12]. Significant levels of tumor hypoxia suggest that esophageal cancers may also experience high levels of replication stress therefore making them sensitive to ATR inhibition [13]. Here, we demonstrate that the addition of the ATR inhibitor VX-970 both chemo and radio-sensitizes esophageal cancer cell lines *in vitro* and most importantly, that this translates to a significant tumor growth delay when combined with radiation in an *in vivo* model.

## Materials and methods

### Cell lines and reagents

OE21, OE33 (both SCC) and FLO-1 (ACA) cells were obtained from PHE culture collections. OE21 and OE33 cells were cultured in RPMI, while FLO-1 were grown in DMEM, all supplemented with 10% FBS. Cells were routinely tested for mycoplasma and found to be negative. Cisplatin, 5-fluorouracil (5-FU), paclitaxel (Sigma–Aldrich) and carboplatin (Tocris Bioscience) were used as indicated in individual experiments.

### Hypoxia treatment

Cell migration assays using the xCELLigence equipment were carried out in a humidified incubator set to 2% O_2_. Other hypoxic treatments were carried out in a Bactron chamber (Shel Lab) at <0.1% O_2_ or in a Don Whitley H35 Hypoxystation at 2% O_2_.

### Immunoblotting

The antibodies used were ATR-T1989 (Millipore), Chk1-S317, Chk1-S345, KAP1-S824 (Cell Signaling Technology), KAP1-S473 (Biolegend), KAP1 (Bethyl/Universal Biologicals Cambridge), Chk1, ATR, β -actin (Santa Cruz Biotechnology), HIF-2α (Novus Biologicals), HIF-1α(BD Biosciences) and GAPDH (Stratech Scientific).

### xCELLigence assay

Real-time monitoring of OE21 cell migration was performed using the xCELLigence RTCA DP instrument with the CIM-Plate 16 (Roche) according to the manufacturer’s instructions. Cells were seeded at a density of 40,000 cells/well and 10% FCS was used as chemo-attractant.

### Colony survival

For the combination of VX-970 and chemotherapeutic drugs, the cells were seeded and 4 h later pre-treated with DMSO or VX-970 for 2 h. Subsequently cisplatin, carboplatin, 5-FU or paclitaxel were added. For the cisplatin treatment the media was unchanged while colonies formed, while for the carboplatin, 5-FU and paclitaxel treatment the medium was replaced with drug-free media after 24 h of treatment. For the combination of radiation and hypoxia, cells were treated with VX-970 and exposed to <0.1% O_2_ for 6 h followed by radiation treatment. In each case colonies of more than 50 formed over a period of 7–10 days. Colonies were stained with crystal violet and the data analyzed as described previously [14].

### Radiation treatment

Cells were irradiated with γ-rays from a Cs-137 irradiator (GSR D1 Gamma-Service Medical GmbH, Germany; Dose rate 1.7 Gy/min). For irradiation in hypoxic conditions, cells were sealed inside the hypoxia chamber in purpose built airtight boxes and then transported to the irradiator. Dosimetry was performed using EBT2 film (ISP Technologies Inc., NJ, USA) irradiated in the position of cells. The exposed EBT2 film strips were scanned and the optical density values corrected as recommended by the manufacturer and converted to dose using a calibration curve obtained from previously scanned film strips, irradiated with a range of known doses using ^60^Co γ-rays.

### Xenograft tumors

All animal procedures were performed in accordance with current UK legislation and were approved by the University of Oxford Biomedical Services Ethical Review Committee, Oxford, UK. OE21 cells were grown as xenograft tumors as previously described [15]. 6–8 week old female CD-1 nude mice (Charles River, UK) were injected subcutaneously into the flank with 5 × 10^6^ OE21 cells in 50% (v/v) matrigel and serum-free RPMI. Animal groups received either vehicle (10% Vitamin E d-alpha tocopherol polyethylene glycol 1000 succinate) or 60 mg/kg of VX-970 orally on 5 subsequent days. 2 groups received a single dose of radiotherapy (10 Gy) 2 h after the second vehicle or VX-970 treatment. An additional 3 animals per each group were harvested one day after radiation for IHC and ATR inhibition analysis. 2 h before the tumor was harvested, mice were injected intraperitoneally with 60 mg/kg of pimonidazole. Hypoxic regions were visualized by pimonidazole staining with hypoxyprobe 1 antibody (clone 4.3.11.3, Hypoxyprobe) after dewaxing and antigen retrieval with 10 mM sodium citrate buffer (pH 6.0). Alternatively sections were stained for 53BP1 (NB100-904, Novus Biologicals) or ATR-T1989 (ABE462, Millipore), all followed by HRP-conjugated secondary antibody incubation. Staining was developed with 3,3′-Diaminobenzidine (DAB, Vector Labs), and nuclei were counterstained with hematoxylin. Images were obtained using an Aperio Scanner (Leica Biosystems).

### Statistical analysis

Statistical analysis was performed using GraphPad Prism 6 software (GraphPad Software Inc.). For the cell migration, colony survival assays and tumor growth analysis the 2-way ANOVA test was used. For the analysis of 53BP1 and ATR-T1989 one-way ANOVA test was used, followed by student t test for each comparison. Tumor growth curves were analyzed with two-way ANOVA between individual groups. *P* values of less than 0.05 considered as significant (^*^P < 0.05; ^**^P < 0.01; ^***^P < 0.001; ^****^P < 0.0001; ns non-significant).

## Results

**Fig. 1:**
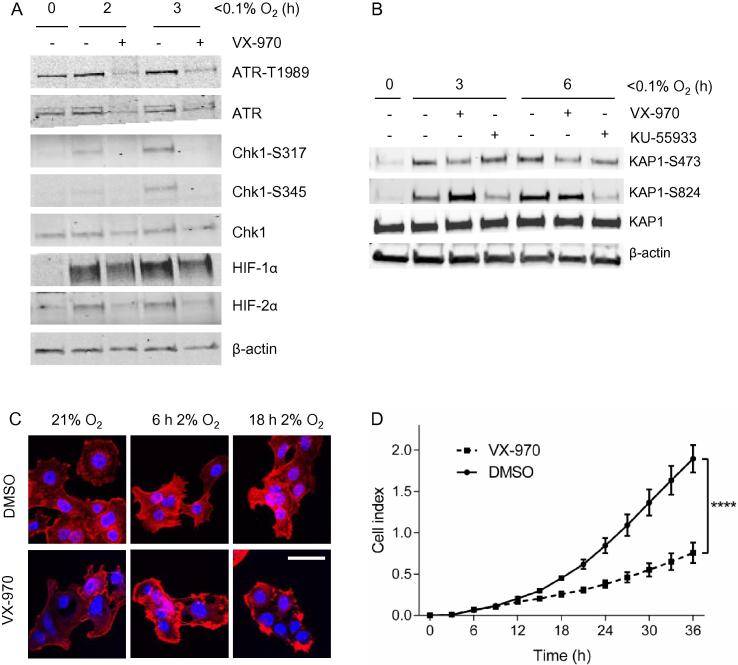
VX-970 inhibits ATR-mediated signaling in OE21 cells exposed to hypoxia. OE21 cells were treated with VX-970 (1 μM) and then exposed to hypoxia (<0.1% O_2_) for the times indicated. Western blotting was then carried out using the antibodies indicated, β-actin was used as a loading control (A). (B) OE21 cells were treated with VX-970 (1 μM) or KU-55933 (10 μM) and then exposed to hypoxia (<0.1% O_2_) for the times indicated. Western blotting was then carried out. (C) OE21 cells were treated with VX-970 (1 μM) and exposed to a milder level of hypoxia (2% O_2_) for the times indicated. Cells were fixed and stained with phalloidin to visualize F-actin (scale bar = 50 μm). (D) OE21 cells migration in response to VX-970 treatment in hypoxia (2% O_2_) was analyzed by xCELLigence. Results shown are mean ± SEM (*n* = 3). Significance: Two-way ANOVA test, ^****^P < 0.0001*.*

To determine if inhibition of ATR using VX-970 inhibits ATR-mediated signaling in esophageal cancer we treated OE21 cells with the inhibitor and then exposed them to hypoxia (<0.1% O_2_) in order to induce ATR activity [16]. As expected an increase in phosphorylated Chk1 at both residues S317 and S345 was observed in response to hypoxia and this was decreased in the presence of VX-970. For the first time, we also observed an induction of phosphorylated ATR at T1989 in response to hypoxia and this was also inhibited by VX-970 (Fig. 1A). In addition, we verified that VX-970 treatment did not inhibit hypoxia-induced ATM mediated signaling by investigating KAP-1 phosphorylation in hypoxia. OE21 cells were treated with either VX-970 or the well-established ATM inhibitor, KU-55933, and exposed to hypoxia. VX-970 decreased the levels of the ATR-Chk1 dependent phosphorylation site S473 on KAP-1 while not effecting S824 (a site phosphorylated predominantly by ATM) (Fig. 1B) [16], [17]. Together, these data verify that VX-970 specifically and effectively inhibits hypoxia-induced ATR signaling in esophageal cancer cells. In accordance with previous reports, we also observed a reduction in HIF-1α stabilization when the ATR inhibitor was present (Fig. 1A) [5], [18]. Interestingly, the level of HIF-2α stabilization in response to hypoxia was also decreased by the addition of VX-970. As HIF is also stabilized at higher oxygen concentrations than those required to induce ATR activity, we exposed OE21 cells to a milder level of hypoxia (2% O_2_) and measured HIF-1α and HIF-2α stability. In both cases these transcription factors were stabilized slightly less in the presence of the ATR inhibitor (Fig. S1). It is unclear if this difference would be significant enough to affect the HIF mediated biological response to hypoxia. However, we noted that the cells treated with VX-970 in mild hypoxia had a more rounded appearance with an accumulation of the actin cytoskeleton at the cell edges (visualized with F-actin staining), in comparison to cells that were treated with DMSO (Fig. 1C). The appearance of the cells suggested to us that they might be less motile in the presence of VX-970 and so this hypothesis was tested using the xCELLigence assay [19]. OE21 cells were incubated in hypoxic conditions (2% O_2_) in the presence of VX-970 or DMSO as a control and cell motility was measured over 36 h (Fig. 1D). Cells became significantly less motile in hypoxia in the presence of the ATR inhibitor. These data are supportive of a role for ATR in HIF stabilization and consequently HIF-mediated motility but do not rule out a HIF-independent effect of ATR on cell migration.

**Fig. 2:**
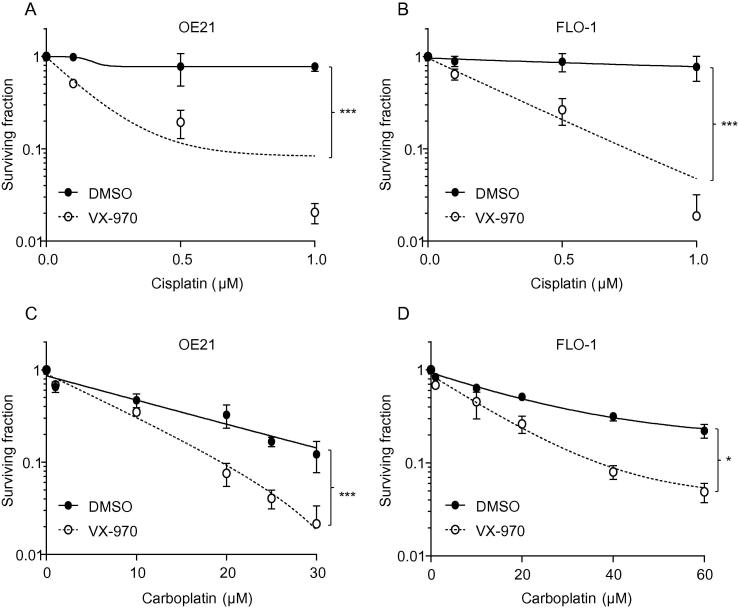
ATR inhibition with VX-970 increases the sensitivity of esophageal cancer cells to cisplatin and carboplatin. OE21 (A) and FLO-1 (B) cells were exposed to a combination of VX-970 (50 nM) and cisplatin as indicated and a colony survival assay carried out. OE21 (C) and FLO-1 (D) were exposed to carboplatin and VX-970 (50 nM) and a colony survival assay carried out. Results shown are mean ± SEM (*n* = 3). Significance: Two-way ANOVA test, ^*^P < 0.05; ^***^P < 0.001*.*

**Fig. 3:**
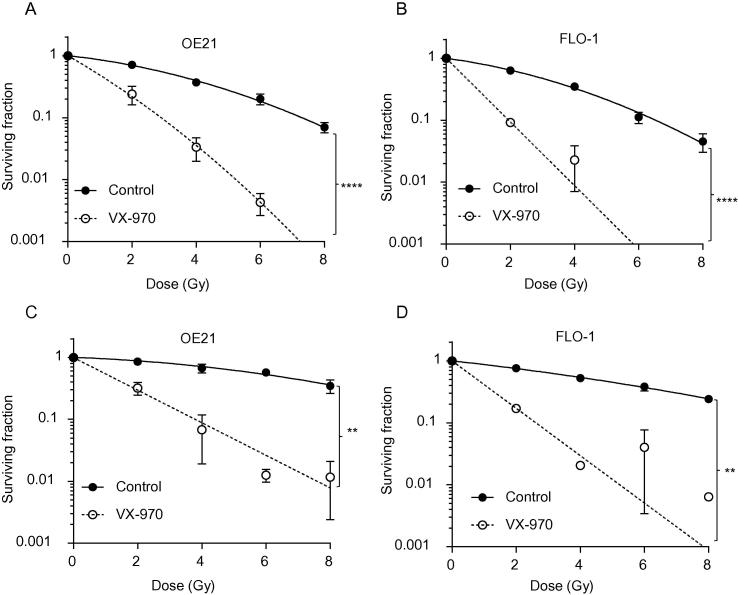
VX-970 treatment increases the sensitivity of esophageal cancer cells to radiation in normoxic and hypoxic conditions. OE21 (A) and FLO-1 (B) cells were treated with VX-970 (50 nM) and then irradiated with the doses indicated followed by a colony survival assay. This experiment was then repeated in hypoxic conditions (<0.1% O_2_) for OE21 (C) and FLO-1 (D) cells. Results shown are mean ± SEM (*n* = 3). Significance: Two-way ANOVA test, ^**^P < 0.01,^****^P < 0.0001*.*

We carried out preclinical testing of VX-970 in combination with standard therapies and in conditions which mimic the tumor microenvironment. Initially, we considered the efficacy of combining VX-970 with cisplatin and carboplatin as both these agents induce DNA damage and are used to treat esophageal cancer. OE21 and FLO-1 cells were exposed to a range of doses of cisplatin or carboplatin plus VX-970 (50 nM) or a DMSO control and colony survival assays were carried out (Fig. 2). In each case the addition of the ATR inhibitor proved to significantly increase the loss of viability observed in response to the chemotherapeutic agent. A similar response was also observed in OE33 cells exposed to cisplatin plus VX-970 (Fig. S2). In contrast, we did not observe a significant increase in loss of viability when VX-970 was used in combination with 5-FU or paclitaxel in OE21 or FLO-1 cells (Fig. S3). Next we investigated the combination of VX-970 and radiation in esophageal cell lines. The addition of VX-970 significantly increased radiation-induced loss of viability as determined by colony survival assay in OE21, FLO-1 (Fig. 3A/B) and OE33 cells (Fig. S4A). To explore this effect further we repeated these experiments in hypoxic conditions to mimic the radiation resistance of tumor cells *in vivo*. OE21, FLO-1 and OE33 cells were significantly radiosensitized by VX-970 in hypoxic conditions (<0.1% O_2_) (Fig. 3C/D, S4B).

**Fig. 4:**
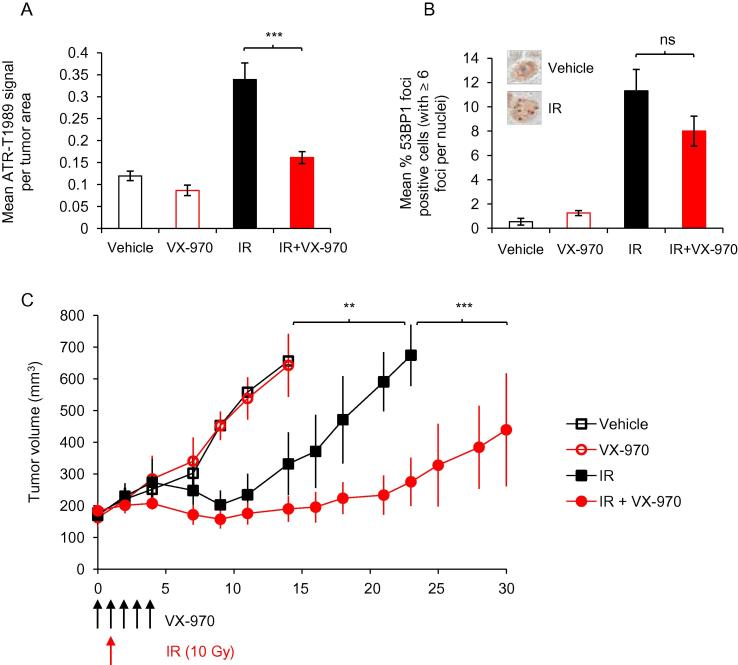
VX-970 treatment improves radiation response *in vivo*. (A-B) OE21 xenografts were collected on day 3 of the VX-970 treatment/one day after radiation (10 Gy), formalin fixed and paraffin-embedded. Immunohistochemical images of phosphorylated ATR-S1989 and 53BP1 foci (examples shown in Fig. S5C and D) were quantified. Bar graph showing the mean ± SEM signal from phospho-ATR-T1989 staining from at least 20 section areas analyzed for each condition (at least 2 tumors per condition) (A). Bar graph showing the mean ± SEM percent of cells per section area positive for 53BP1 foci (>6 foci per nuclei, examples shown in the insert boxes) from at least 20 section areas analyzed for each condition (at least 2 tumors per condition) (B). One-way ANOVA test, followed by student t test for each comparison shows statistical significance; ^***^P < 0.001, ns non-significant. (C) Tumor growth rates were determined for the 4 tumor groups indicated. Graph shows mean ± SEM (*n* = 4 in each group) analyzed with a two-way ANOVA test between individual groups; ^**^P < 0.01, ^***^P < 0.001.

Subsequently, we grew xenograft tumors from OE21 cells and investigated the combination of ATR inhibitor and radiation *in vivo*. OE21 cells formed tumors, which upon analysis of pimonizadole staining could be seen to contain widespread regions of hypoxia (Fig. S5A). Representative tumors from each treatment group were harvested one day after radiation and protein extracts prepared to verify that the ATR inhibitor had been effective. A clear decrease in the levels of both phosphorylated ATR and Chk1 were observed in a tumor treated with VX-970 (Fig. S5B). VX-970 mediated inhibition of ATR-T1989 was also determined by immunohistochemistry on harvested tumor sections (Fig. 4A and S5C). In addition we stained tumor sections for 53BP1 and observed the expected increase in 53BP1 foci in tumors treated with radiation. However, using the tumors harvested and analyzed at this point in the treatment schedule there was no significant VX-970 mediated decrease in 53BP1 foci formation (Fig. 4B and S5D). Tumor volume was measured over a thirty day period and demonstrated a significant growth delay in those treated with VX-970 and radiation compared to either agent alone (Fig. 4C). Further growth delay was observed over 50 days in the combined treatment group (Fig. S6).

## Discussion

We have shown in three esophageal cancer cell lines (two SCC and one ACA) that the addition of the ATR inhibitor VX-970 increases loss of viability in response to cisplatin, carboplatin and radiation. In the case of radiation we also demonstrate that this combination is still effective in hypoxic conditions which mimic the radiation-resistant fraction of esophageal tumors. Furthermore, we show that VX-970 radiosensitizes tumors grown from the OE21 cell line *in vivo*. This preclinical evidence strongly suggests that the addition of VX-970 could be an effective strategy to improve treatment response to esophageal cancer. In addition, we also highlight the possibility that ATR inhibition may also be beneficial due to roles outside of the DNA damage response. The data presented suggest that effects on cell motility and HIF-1/2 signaling should be evaluated during further preclinical testing of ATR inhibitors. One possible hypothesis to explain the decreased motility observed is that HIF/VEGF dependent signaling to Rho GTPases is reduced as a result of ATR inhibition [20]. Our study also suggests that ATR-T1989 and KAP1-S473 should be further investigated as potential biomarkers for inhibition of ATR.

Understanding biology is key to the successful development of new treatment strategies for esophageal cancer. The only biomarker driven therapy in clinical practice to date is trastuzumab, a humanized monoclonal antibody against HER2 [21] which is amplified in 15–25% of adenocarcinomas of the esophagus; future treatment strategies should increasingly incorporate drugs that exploit aberrations in specific signaling pathways (in this case, ATR inhibition in a p53 mutated tumor). Radiotherapy combined with cisplatin and a fluoropyrimidine (5-FU or capecitabine) is the treatment of choice for patients with localized disease who are unsuitable for surgery and it is being increasingly considered as an alternative to surgery in patients with SCC histology. Survival outcomes from esophageal cancer remain poor with less than 20% of patients being suitable for surgery due to disease extent or co-morbidities; even in surgically treated patients median survival is reported to be 24 months [22]. In the UK the SCOPE1 trial failed to show benefit from adding cetuximab to standard dCRT regimen, but did show an unprecedented survival in the standard arm [23]. More recently, the combination of carboplatin with paclitaxel with radiation has been shown to be an effective modality in the pre-operative setting with one phase III trial showing a doubling of median survival from 24 to 48 months, with low incidence of grade 3–4 treatment associated toxicity and no increase in peri-operative mortality [24]. These studies suggest that further enhancing radiosensitivity may potentially result in improving cure rates in this disease. Our data show that the addition of an ATR inhibitor to these regimens can be a potentially effective strategy to improve cure rates in this disease or spare morbid surgery from which patients may take up to 2 years to regain their former quality of life [25]. The combination of VX-970 with radiotherapy alone and with cisplatin-capecitabine based chemoradiation will be tested in CHARIOT, a phase I trial, which will open shortly in the UK (EudraCT number: 2015-003965-27).

## Conflict of interest

None.
