# Supplementary material for: Preclinical testing of an Atr inhibitor demonstrates improved response to standard therapies for esophageal cancer
Source: Radiother Oncol. 2016 Nov;121(2):232–8. doi: 10.1016/j.radonc.2016.10.023 (PMC5154234; doi:10.1016/j.radonc.2016.10.023)
Supplement: Supplementary data 1 — Supplementary Figs. 1–6. [file mmc1.pptx]

## Slide 1
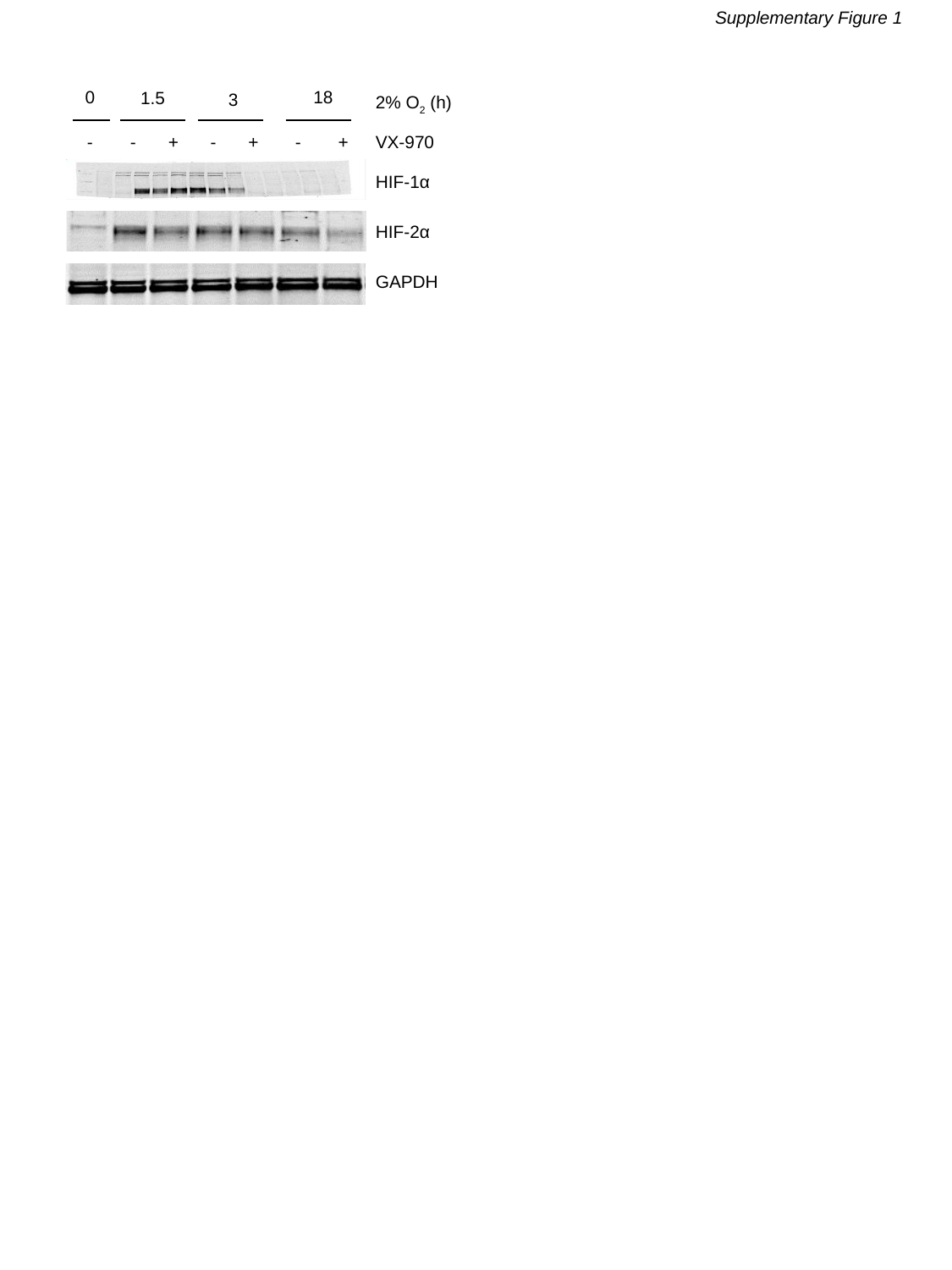

Supplementary Figure 1
18
0
1.5
3
2% O2 (h)
-
-
+
-
+
-
+
VX-970
HIF-1α
HIF-2α
GAPDH

## Slide 2
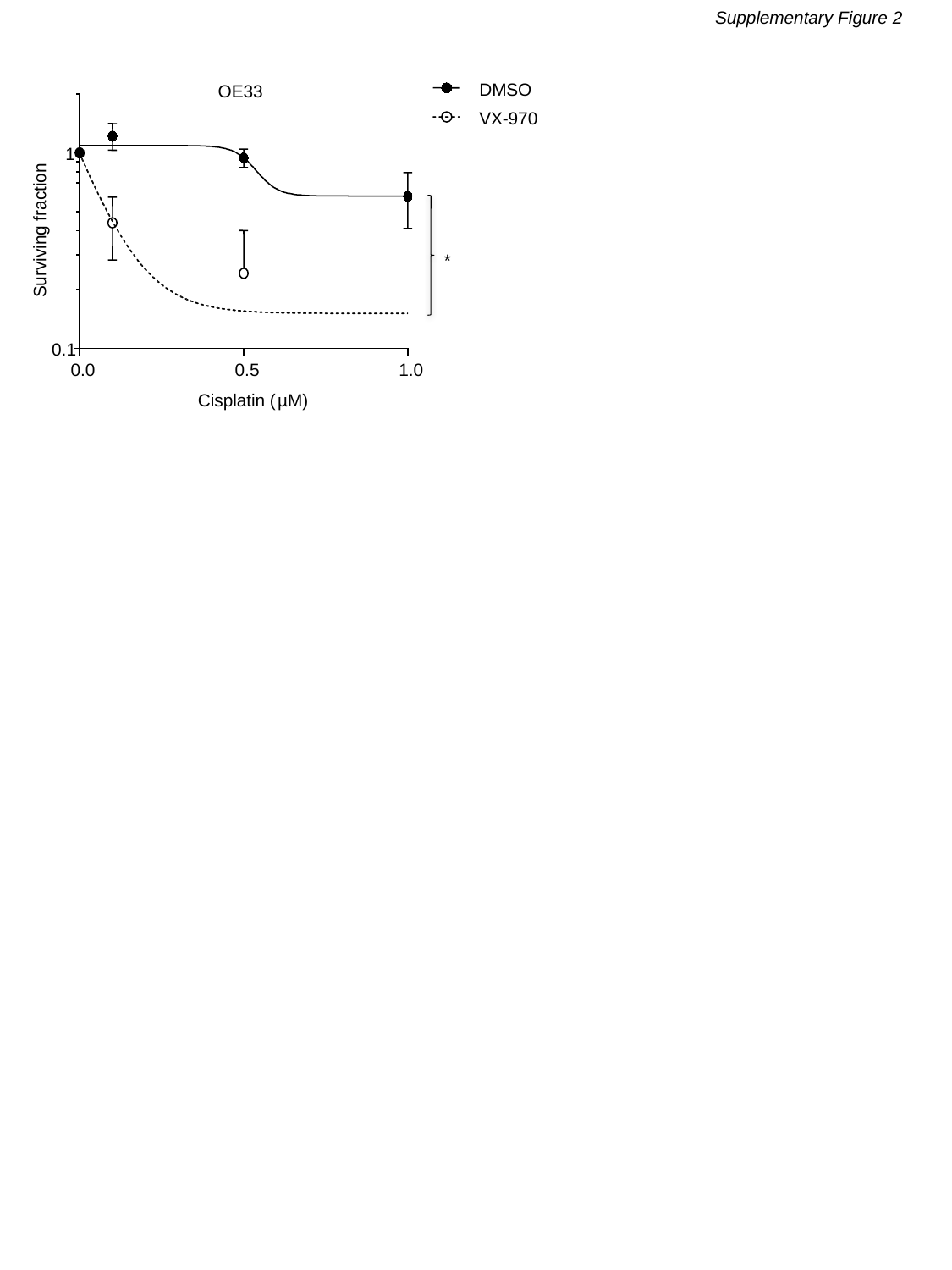

Supplementary Figure 2
OE33
DMSO
VX-970
1
0.1
0.0
0.5
1.0
Surviving fraction
*
Cisplatin (
µM)

## Slide 3
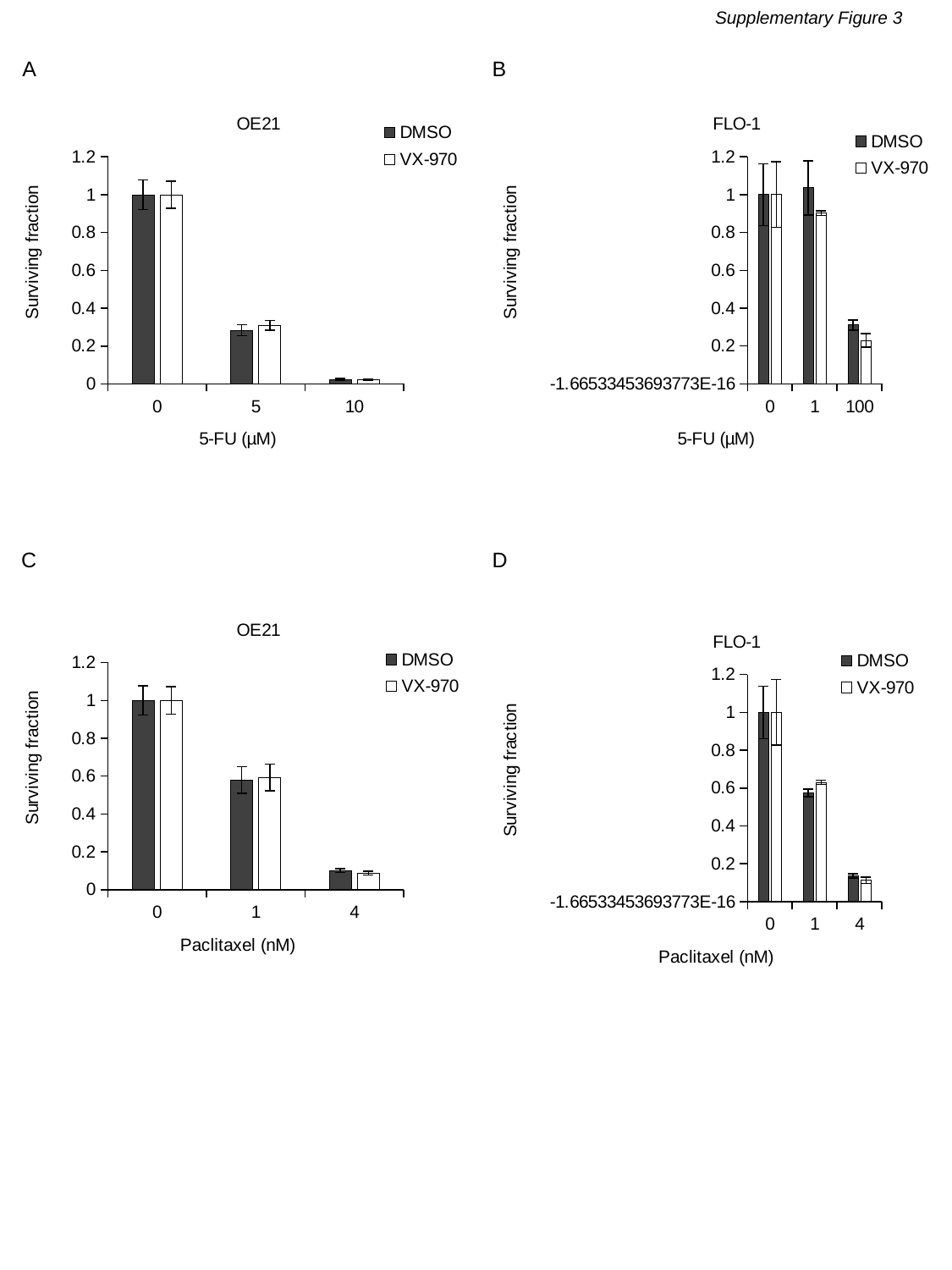

Supplementary Figure 3
B
A
### Chart: OE21
| Category | | |
|---|---|---|
| 0 | 0.9999999999999999 | 1.0 |
| 5 | 0.2835051546391752 | 0.30952380952380953 |
| 10 | 0.022909507445589918 | 0.021645021645021648 |
### Chart: FLO-1
| Category | | |
|---|---|---|
| 0 | 1.0 | 1.0 |
| 1 | 1.0355329949238579 | 0.9028571428571429 |
| 100 | 0.31091370558375636 | 0.23 |C
D
### Chart: OE21
| Category | | |
|---|---|---|
| 0 | 0.9999999999999999 | 1.0 |
| 1 | 0.579037800687285 | 0.5930735930735931 |
| 4 | 0.10008591065292094 | 0.08712121212121211 |
### Chart: FLO-1
| Category | | |
|---|---|---|
| 0 | 1.0 | 1.0 |
| 1 | 0.575242718446602 | 0.63 |
| 4 | 0.1359223300970874 | 0.11285714285714286 |

## Slide 4
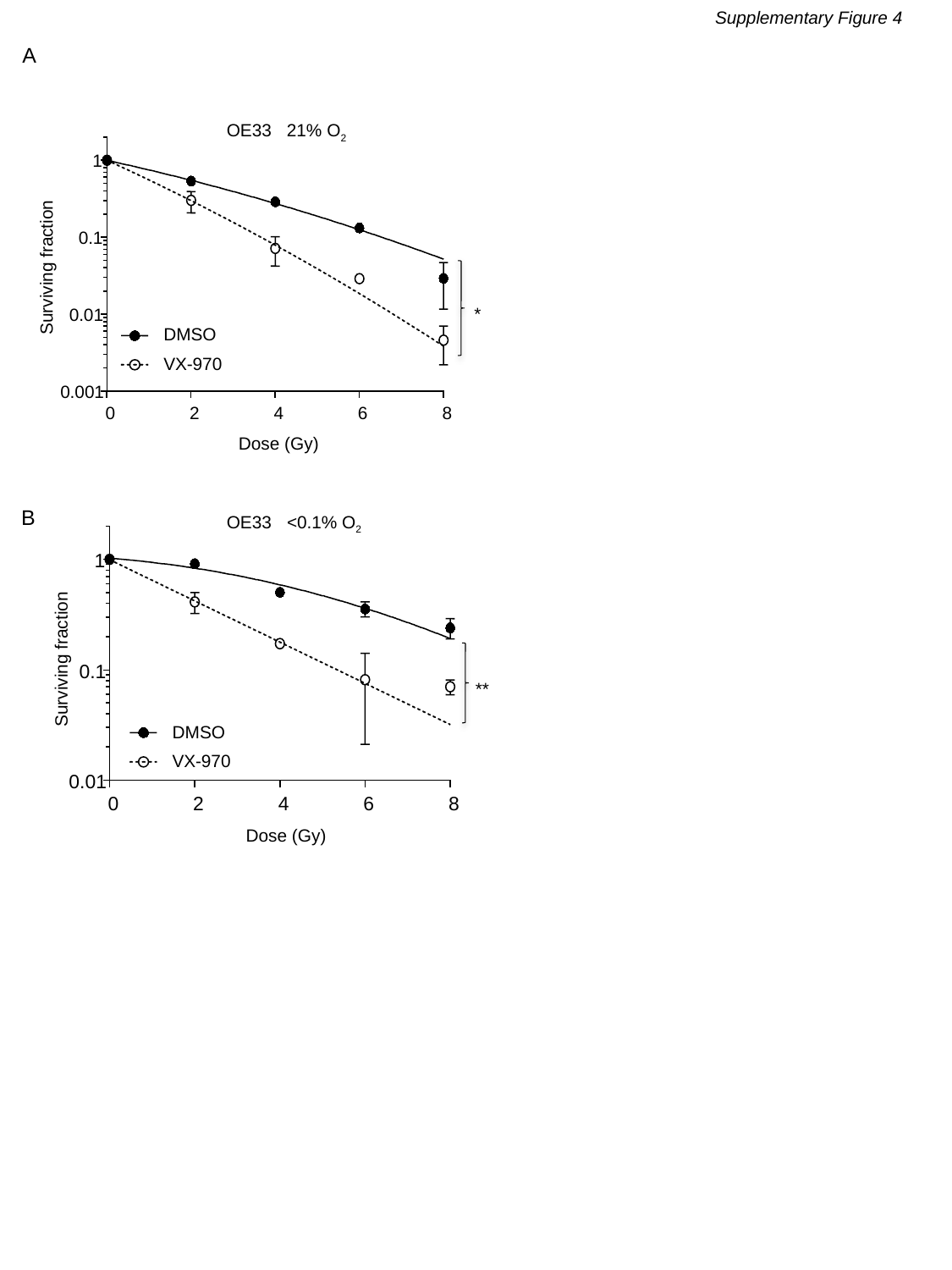

Supplementary Figure 4
A
OE33
21% O2
1
0.1
0.01
0.001
0
2
4
6
8
Surviving fraction
*
DMSO
VX-970
Dose (Gy)
B
OE33
<0.1% O2
1
0.1
0.01
0
2
4
6
8
Surviving fraction
**
DMSO
VX-970
Dose (Gy)

## Slide 5
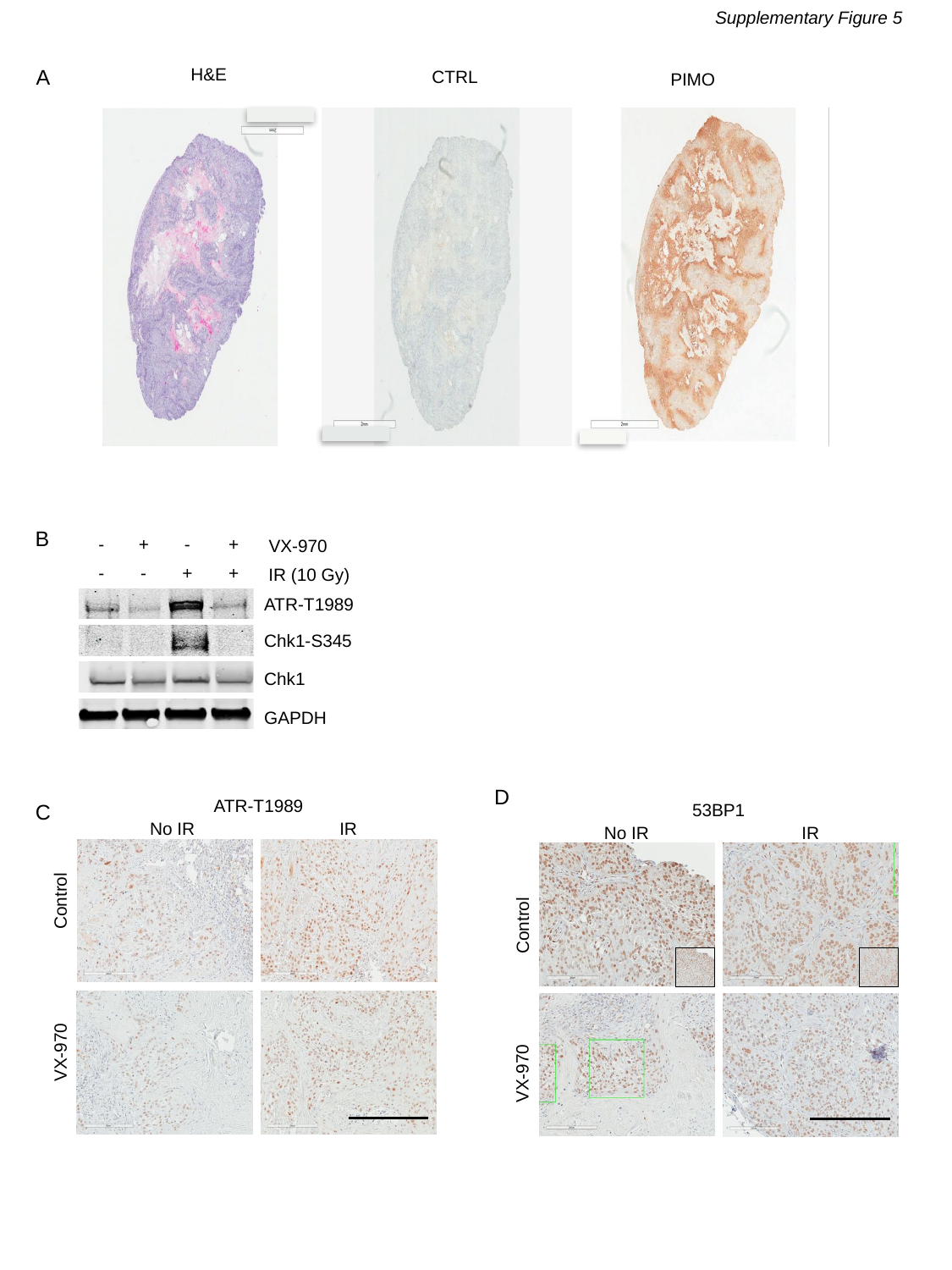

Supplementary Figure 5
H&E
CTRL
PIMO
A
B
-
+
-
+
VX-970
-
-
+
+
IR (10 Gy)
ATR-T1989
Chk1-S345
Chk1
GAPDH
D
ATR-T1989
IR
No IR
Control
VX-970
C
53BP1
IR
No IR
Control
VX-970

## Slide 6
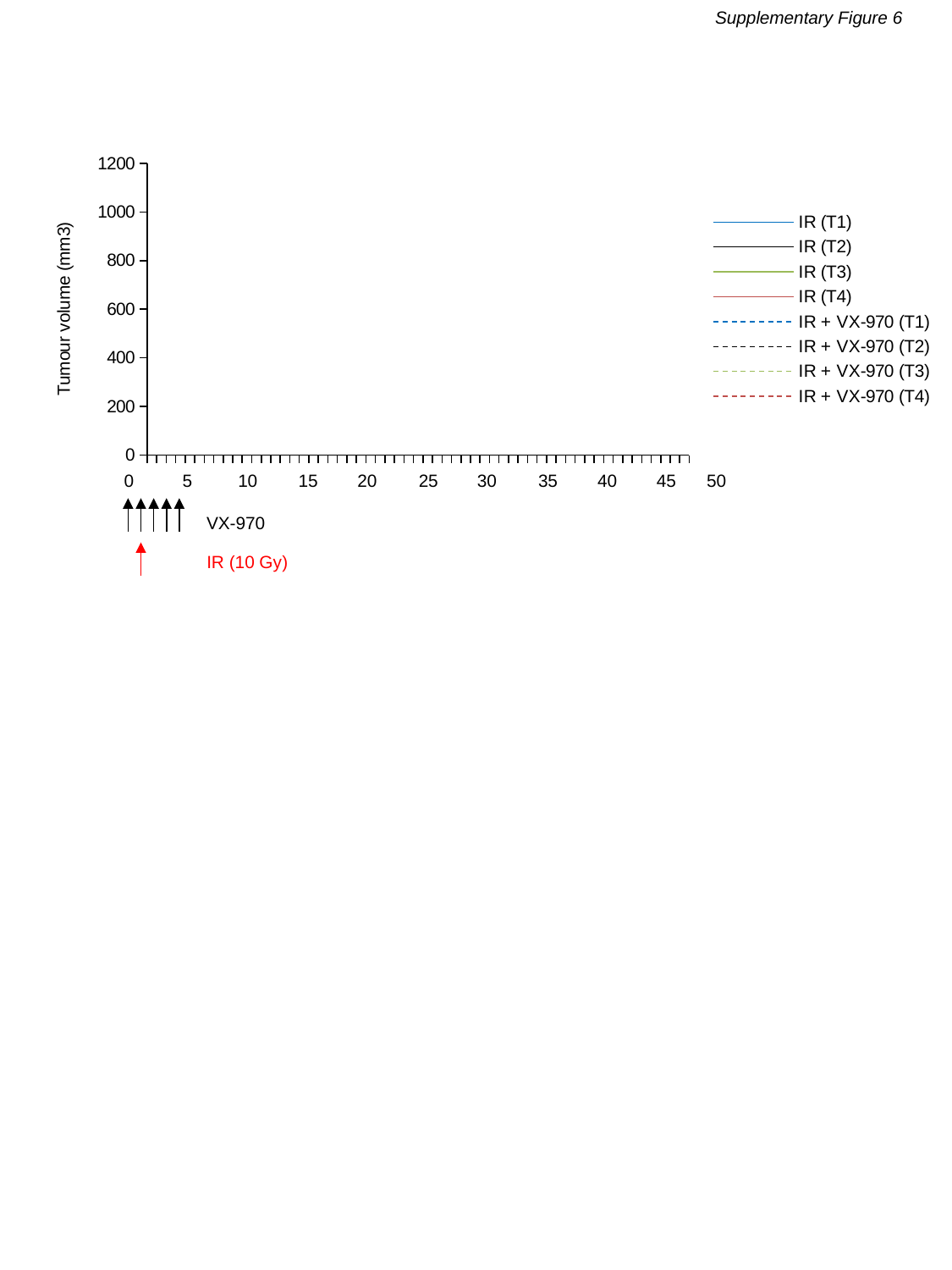

Supplementary Figure 6
[unsupported chart]
0
5
10
15
20
25
30
35
40
45
50
VX-970
IR (10 Gy)
