# Supplementary material for: Preclinical testing of an Atr inhibitor demonstrates improved response to standard therapies for esophageal cancer
Source: Radiother Oncol. 2016 Nov;121(2):232–8. doi: 10.1016/j.radonc.2016.10.023 (PMC5154234; doi:10.1016/j.radonc.2016.10.023)
Supplement: Supplementary data 2 — Legends for Supplementary figures. [file mmc2.docx]

Leszczynska et al.,

Supplementary Figure legends

Fig. S1. ATR inhibition with VX-970 moderately impairs HIF-1α and HIF-2α stabilization under mild hypoxic conditions. OE21 cells were treated with VX-970 (1 μM) and exposed to 2% O_2_ for the times indicated. Western blotting was then carried out using the antibodies indicated, β-actin was used as a loading control.

Fig. S2. ATR inhibition with VX-970 increases the sensitivity of OE33 cells to cisplatin. OE33 cells were exposed to a combination of VX-970 (50 nM) and the indicated doses of cisplatin, followed by a colony survival assay. Results shown are mean ± SEM (n = 3). Significance: Two-way ANOVA test, **P* < 0.05*.*

Fig. S3. ATR inhibition with VX-970 did not increases the sensitivity of esophageal cancer cells to 5-FU or paclitaxel. OE21 (A) and FLO-1 (B) cells were exposed to a combination of VX-970 (50 nM) and 5-FU as indicated and a colony survival assay carried out. OE21 (C) and FLO-1 (D) were exposed to paclitaxel and VX-970 (50 nM) and a colony survival assay carried out.

Fig. S4. ATR inhibition with VX-970 increases the sensitivity of OE33 cells to radiation in normoxic and hypoxic conditions. OE33 cells were treated with VX-970 (50 nM) and exposed to 6 hours of 21% O_2_ (A) or <0.1% O_2_ (B) and then irradiated under the same oxygen tensions as indicated. A colony survival assay was then carried out. Results shown are mean ± SEM (n = 2). Significance: Two-way ANOVA test, **P* < 0.05, ***P* < 0.01.

Fig. S5. Immunohistochemistry of OE21 xenografts showing tumor hypoxia, 53BP1 and phosphorylated ATR-T989. (A) OE21 xenograft tumors were stained with hematoxylin and eosin (H&E) for morphology or with anti-pimonidazole (PIMO) to visualize hypoxic regions. Staining with anti-mouse secondary antibody alone is shown as a negative control (CTRL). (B) Protein extracts were prepared from tumors harvested on day 3 of the VX-970 treatment/one day after radiation (10 Gy) and used to determine efficacy of ATR inhibition. Samples were western blotted as indicated. (C) and (D) representative images of immunohistochemical staining of ATR-T1989 and 53BP1, respectively, in OE21 xenografts, which were treated and collected as in (B). All sections were counterstained with hematoxylin. Scale bars, 100 µm.

Fig. S6. Extended growth curves of individual OE21 tumors from Fig. 4 treated with VX-970 (60 mg/kg) and radiotherapy (10 Gy).
